# Impact of Reducing Sitting Time in Women with Fibromyalgia and Obesity: A Randomized Controlled Trial

**DOI:** 10.3390/ijerph18126237

**Published:** 2021-06-09

**Authors:** Beatriz Rodríguez-Roca, Fernando Urcola-Pardo, Ana Anguas-Gracia, Ana Belén Subirón-Valera, Ángel Gasch-Gallén, Isabel Antón-Solanas, Ana M. Gascón-Catalán

**Affiliations:** 1 Department of Physiatry and Nursing, Faculty of Health Sciences, University of Zaragoza, C/Domingo Miral s/n, 50009 Zaragoza, Spain; brodriguez@unizar.es (B.R.-R.); furcola@unizar.es (F.U.-P.); aanguas@unizar.es (A.A.-G.); subiron@unizar.es (A.B.S.-V.); agascon@unizar.es (A.M.G.-C.); 2 Research Group Water and Environmental Health (B43_20R), University Institute of Research in Environmental Science of Aragón, University of Zaragoza, 50009 Zaragoza, Spain; 3 Research Group Safety and Care (GIISA0021), Institute of Research of Aragón, 50009 Zaragoza, Spain; 4 Research Group Sector III Healthcare (GIIS081), Institute of Research of Aragón, 50009 Zaragoza, Spain; 5 Research Group Nursing Research in Primary Care in Aragón (GENIAPA) (GIIS094), Institute of Research of Aragón, 50009 Zaragoza, Spain

**Keywords:** sitting time, sedentary behavior, fibromyalgia, obesity

## Abstract

Background: Sitting time has negative effects on health, increasing the risk of obesity, osteoporosis, diabetes, and cancer. Thus, primary health care education interventions aimed to reduce sitting time and sedentary behavior could have beneficial effects on people’s health and wellbeing. The purpose of this study was to assess the effectiveness of an intervention based on reducing sitting time to decrease cardiometabolic risk on a sample of women diagnosed with fibromyalgia and moderate obesity. Methods: Randomized controlled trial to evaluate the effectiveness of an intervention to decrease cardiometabolic risk in 84 participants. Sedentary behavior was monitored using an accelerometer before and at 3-month follow-up. Results: Compared with the control group, body mass index decreased, and the number of steps taken increased, in the intervention group 3 months after the intervention. No significant differences were found in the rest of the variables measured. Conclusion: The intervention group decreased sitting time after the intervention. Group activities and support from primary care may be useful to improve treatment adherence. RCT registration: NCT01729936.

## 1. Introduction

Fibromyalgia (FM) is a chronic disorder characterized by chronic widespread musculoskeletal pain [1] and accompanied by fatigue, cognitive disturbances, psychiatric, and multiple somatic symptoms [2]. FM has an unknown etiology and uncertain pathophysiology. As suggested by ongoing research, FM is considered to be a pain regulation disorder and often classifies as a form of central sensitization syndrome [3]. Due to the nature of its symptoms, as well as its chronic nature, patients diagnosed with FM tend to engage in sedentary behavior (SB) [4]. SB occurs when “the energy expended is less than or equal to 1.5 metabolic equivalents (MET), keeping the posture of sitting or lying down” [5]. SB is considered one of the major public health problems and it is a risk factor for the development of noncommunicable diseases [6] including obesity [7], diabetes [8], certain types of cancer [9], and sleep disturbances [10]. According to van der Ploeg [11] and Pines [12], SB shortens life expectancy and increases the risk of mortality independently of physical activity. However, according to other authors [13], the risk of death associated with prolonged sitting time (ST) can be reduced by moderate physical activity for 60 to 75 min a day.

There is no curative treatment for FM. Symptom management strategies include pharmacological and nonpharmacological treatments aimed at alleviating pain, increasing restorative sleep, improving physical function, and, ultimately, improving quality of life [14]. As demonstrated by previous research, physical activities including aerobic exercise [15], strength exercises [16], pool activities [17], and daily steps [18] have a beneficial effect on people with FM. Specifically, physical activity can reduce the perception of pain and depression, and can improve the quality of sleep and self-management in patients with FM [18,19]. However, despite the evidence, women with FM tend to spend less time engaging in moderate and high intensity physical activity [20].

Previous studies on SB in different population groups, namely office workers [21], patients with chronic conditions [22], and even children [23], have found an association between reduced SB and positive health outcomes. In the population of patients with FM, a positive correlation has been established between reduced SB and impact of the disease on quality of life [19], pain, and fatigue [24]. However, as outlined above, patients with FM are significantly less active than sedentary healthy controls. Whereas ST seems to be consistently associated with symptoms of FM [25], there is insufficient evidence about the effects of reducing ST on health outcomes of people with FM who are overweight or moderately obese.

In Spain, people with FM are frequently managed in the community by primary care nurses and physicians [26,27]. Primary healthcare providers are responsible for implementing health promotion programs, which can have a significant impact on patients’ lifestyle [28]. To our knowledge, there are no previous studies on health promotion interventions aimed at reducing ST in patients with FM in our context. Our initial hypothesis was that reducing sitting time through a primary care health education intervention would reduce cardiometabolic risk in women with FM who were overweight or moderately obese. Therefore, the aim of this study was to evaluate the impact of a primary care health education intervention to reduce ST on cardiometabolic risk in a sample of patients diagnosed with FM and who were overweight or moderately obese.

## 2. Materials and Methods

### 2.1. Design

We present the results from a randomized controlled trial to reduce daily hours of ST in overweight and obese women with FM.

### 2.2. Participants

The participants were recruited from two primary care centers in the city of Zaragoza (Spain) from September 2013 to September 2014. A total of 494 women who had a diagnosis of FM and were moderately obese or overweight were contacted via telephone by a researcher and qualified nurse (B.R.-R.), who explained the purpose of the investigation and invited them to take part in the study. If they accepted, they were given an appointment with the same researcher at their primary care center, where the selection criteria were carefully reviewed, the aim and procedures of the investigation were clearly explained, the consent form was signed (if applicable), and the participants were given an accelerometer with the aim of assessing and recording SB.

The inclusion criteria to participate in this study were: (1) being female, (2) having a formal diagnosis of FM, (3) being 25–65 years old, (4) having a body mass index (BMI) of 25–34.9 kg/m^2^, and (5) spending more than 6 h a day sitting, as measured by the Marshall Sitting Questionnaire (MSQ) in its Spanish version [29]. We excluded individuals with contraindications to regular physical activity including osteoarticular diseases, advanced heart disease, severe walking difficulties, cancer on treatment, individuals who were not able to communicate in Spanish, and those who had an unstable demographic situation.

**Figure 1:**
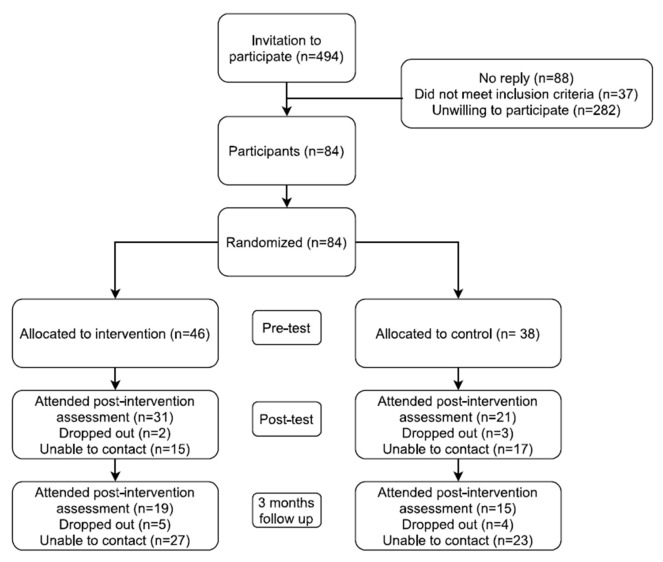
Flowchart of the recruitment process and RCT design.

**Figure 2:**
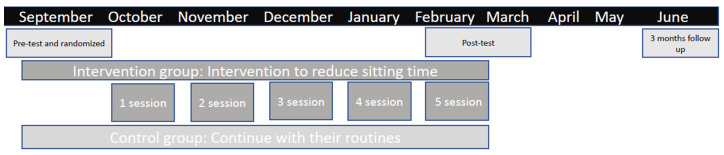
Timeline of study procedures.

Our rejection rate was high, with 282 women refusing to take part in the study. In addition, 88 women could not be contacted (a researcher gave them a phone call three times at different times and there was no reply) and 37 were excluded due to not meeting the selection criteria. A final sample of 84 participants was recruited to participate in the study; 46 were randomized to the intervention group and 38 to the control group following a single-blind randomization method. Unfortunately, only 31 women in the intervention group and 21 in the control group, and 19 in the intervention group and 15 in the control group attended the first and second post-intervention assessments, respectively. Figure 1 shows a flowchart of the recruitment process and RCT design; Figure 2 shows a timeline of the study procedures.

### 2.3. Description of the Intervention

The patients wore the accelerometer constantly for a week before the second appointment. A week after the initial meeting, the participants attended a second, face-to-face appointment with the same researcher (B.R.-R.). During this second appointment, the participants were given the opportunity to ask questions, pretest measurements were taken, the accelerometer was removed, and the participants were randomized to either the intervention or the control group. Patients randomized to the intervention group received a 6 month health education intervention consisting of five face-to-face or telephone appointments, depending on the patient’s preference. The appointments lasted an average of 30 min, and all the patients in the intervention group who completed the process (*n* = 19) attended the five appointments (nonattendance was a reason for exclusion). During these appointments, the researcher and the participants jointly identified opportunities to decrease ST and planned activities in order to decrease SB, namely using the stairs, getting off the bus one stop earlier, standing up and walking after finishing a book chapter, etc. An effort was made to help the patients integrate these activities into their daily routine.

The participants assigned to the control group were encouraged to continue with their daily activities as usual.

The same parameters were measured in the intervention and the control group.

### 2.4. Data Collection

Data were collected during a personal interview before the intervention, immediately after the intervention, and at 3-month follow-up.

The following information was collected by a research nurse through face-to-face interviews with the participants at the beginning of the study. The participants completed a questionnaire of sociodemographic and clinical variables designed ad hoc, including age, marital status, employment status, and educational level. The clinical and anthropometric variables were measured thrice by the same research nurse at the beginning of the study, after the intervention, and at 3-month follow-up. We used a Seca 770 scale to measure weight and height and calculate BMI. The abdominal perimeter and tricipital fold were measured using the same tape measure. Blood pressure was also measured thrice using the same electronic sphygmomanometer; measurements were taken three times and the average values were calculated in order to obtain the final measure.

Biochemical variables, triglycerides, total cholesterol, HDL, LDL, and glucose were measured through a blood test performed at the primary care center.

The MSQ in its Spanish version [29] was used to measure the total number of hours of ST (sedentary behavior) prior to recruitment. This questionnaire comprises 20 items that assess time spent sitting (hours and minutes) on weekdays and weekends in five domains: (a) while travelling to and from places (e.g., work, shops); (b) while at work; (c) while watching television; (d) while using a computer at home; and (e) at leisure, not including watching television (e.g., visiting friends, movies, eating out). ST was considered prolonged if it was over 6 h a day; thus, patients whose ST was less or equal to 6 h a day were excluded (*n* = 37) from this study according the criterion established by Martinez-Ramos et al. [30].

We used a 3M accelerometer developed by PAL Technologies Ltd. in order to measure energy expenditure, hours spent sitting and lying down, changes in position, and number of steps taken. The participants were instructed to wear the activPAL™ (PAL Technologies Ltd., Glasgow, UK) on their thigh for 7 consecutive days. They were asked to remove it only during aquatic activities. When the patient’s activPAL™ was removed (7 days later), the data were downloaded all at once. All the records comprising measurements taken through a period of at least 6 days were considered valid and included in the analyses. The activPAL™ was programmed and used by the participants in both groups throughout two one-week periods: (1) before the intervention, and (2) 3 months after the intervention.

### 2.5. Data Analysis

As per protocol analysis [30], descriptive statistics was used to describe the sample using frequency, percentage, mean, and standard deviation (SD) as appropriate. We analyzed the association between categorical variables using a chi-square test. The Shapiro–Wilk test was used to test for normality of the data. The Kruskal–Wallis test was applied for statistical comparison of quantitative variables that followed a normal distribution, and the ANOVA test was used for those that followed a normal distribution. In order to estimate the effect of the health education program to reduce ST, the results from the pretest and the posttest measures were compared using the nonparametric Wilcoxon test and Friedman test for related means. The Epidat 4.2 program was used to calculate the sample size. Taking 494 people as the total population diagnosed with FM registered at the two primary care centers where the study was carried out, and assuming that the Spanish average BMI is 28.6 in this population, with a standard deviation of 5.1 [31], a maximum error of 3%, and a confidence interval of 95%, we would need a population of 84 people. Drop-out analyses were calculated; the response rate was 17% and the completion rate was 40% [32,33].

Data codification, processing, and analysis were completed using the statistical software Statistical Package for the Social Science (SPSS version 22 for Windows, IBM Corp., Chicago, IL, USA), accepting a level of significance of *p* < 0.05.

### 2.6. Ethical Considerations

This study was reviewed and approved by the Clinical Research Ethics Committee of Aragón (C.P-C.I. PI12/00121) prior to the start of this investigation. The investigation adhered to the principles outlined in the Declaration of Helsinki [34]. All the participants included in the study were informed about the study aims and procedures and gave their informed consent to participate.

## 3. Results

The mean age of our participants was 55 years. Over a third of the participants in both groups were employed. Three quarters of the women in the control group, and just over 60% of the women in the intervention group, were married. Only a minority of our participants were educated to university degree level. No significant differences were found between the women in the intervention group and those in the control group, except for their level of education. The participants’ perception of ST (hours) throughout the day was 6.15 (SD 3.46), as measured by the MSQ (Table 1).

Table 2 presents the intergroup differences between participants’ anthropometric, clinical, and biochemical variables, and activPAL™ values before and after the intervention and at 3-month follow-up. We found no statistically significant differences between the intervention and the control groups at baseline, with the exception of BMI (*p* = 0.017), tricipital fold (*p* < 0.001), triglycerides (*p* = 0.053), and glycemia values (*p* = 0.006). There were no significant differences in any of the variables recorded by activPAL™, and the average time spent sitting was 10.08 (SD 2.79) hours per day. There were no significant differences between the groups immediately after the intervention, with the exception of changes in the position (*p* = 0.029). At 3-month follow-up, we observed a significant reduction in ST in the intervention group compared with the control group (*p* = 0.048). No other significant differences between groups were found at this stage. The Friedman test was used to compare the dependent variables measured at the beginning and at the end of the study between groups (interaction *p*-value). We found that the patients in the intervention group increased the number of steps taken (*p* = 0.000) and had a lower BMI (*p* = 0.000) by the end of the study (Table 2).

Intragroup differences in the control and intervention group at baseline and at 3-month follow-up are shown in Table 3. In the control group, significant differences were observed in BMI (*p* < 0.001) and MET (*p* = 0.021) values before and 3 months after the intervention. We found a significant decrease in the intervention group’s BMI (*p* < 0.001) and diastolic blood pressure (*p* = 0.018) 3 months after the intervention.

## 4. Discussion

This RCT evaluated the impact of a primary care health education intervention to reduce ST on cardiometabolic risk in a sample of patients diagnosed with FM and who were overweight or moderately obese. With regard to the sociodemographic characteristics of our sample, we found that only a minority of our participants had university level studies. At 3-month follow-up, sitting time and diastolic blood pressure decreased in the intervention group compared to the control group. In addition, by the end of the study, the women in the intervention group increased the number of steps taken and reduced their BMI.

ST in our population was similar to that observed in patients with rheumatic diseases and was longer than ST in the general adult population [5,35]. This is not surprising, as people with FM do experiment similar symptoms to patients with rheumatic conditions, including pain and fatigue [36,37].

The results from the MSQ were similar in both groups after randomization, indicating that all of our participants had a similar perception of their SB. We compared the results from the MSQ and the objective measurement of ST by means of the activPAL™ and observed a significant difference between perceived and real ST. This implies that our participants underestimated their ST daily [24,38]. This is in agreement with previous studies [38,39] in a similar population, which suggest that ST values increase when measured objectively through accelerometers, as opposed to subjectively through self-administered questionnaires.

Surprisingly, daily ST was reduced slightly in the control group 6 months after recruitment. This may have been due to the Hawthorne effect [40], whereby knowledge of the purpose of the study may have encouraged the participants in the control group to reduce their ST and increase their daily physical activity. Nevertheless, this reduction in ST in the control group was short-lived as it was not maintained at 3-month follow-up. In contrast, a significant reduction in ST was observed in the intervention group at 3-month follow-up. However, our participants’ SB after the intervention was still prolonged. This may be due to a number of reasons, including the nature of the intervention itself. Specifically, the participants did not wear an accelerometer during the intervention period and, therefore, they had no means to objectively evaluate their SB. It is possible that routinely using an objective measurement of physical activity such as the activPAL™ may contribute to raise awareness of SB in this population and, thus, help patients to gradually increase their level of physical activity and decrease ST. Another possible explanation for the limited impact of the intervention on the participants’ SB is their actual willingness to make changes to their daily habits. We did not take into account the participants’ pre-disposition to make changes. It is likely that, despite agreeing to take part in our study, some of our participants were not ready to change or, as proposed by Prochaska and Diclemente [41], were in a precontemplative stage. We recommend that the participants’ readiness to change is assessed prior to engaging in activities involving a change in daily habits, as it may have an impact on the effectiveness of the intervention.

A slight decrease in BMI and in diastolic blood pressure was observed in the intervention group at 3-month follow-up. However, the impact of the intervention on modifiable cardiometabolic risk factors was not as large as expected. Patients with FM may find it hard to maintain an adequate level of physical activity due to symptoms such as general fatigue and widespread pain. In fact, there is an association between tiredness and inability to perform the activities of daily living in our population [4]. This may result in an increase in BMI [2] and the development of comorbidities including obesity [42], anxiety, and depression [43] in this population. In view of our results, we suggest that interventions of this kind are complemented with other activities which contribute to the reduction of SB. For example, patients with FM could be integrated in regular group activities led by an interdisciplinary healthcare team. Group activities can be an effective method to motivate patients whilst establishing social relationships that could improve their mood and disease management [44,45].

We recommend that interventions aimed at decreasing ST in the population of adults with FM are individualized and tailored to the needs and characteristics of each patient, are supervised by a health professional regularly, and, if possible, are complemented with group activities in order to increase patient motivation and social support [46]. Future studies in this area should analyze whether regularly and objectively measuring physical activity in this population, by means of an accelerometer, improves the patients’ response and adherence to interventions aimed at reducing SB.

### Limitations

Modifying daily habits is not an easy task [47,48,49]. Although most of our patients alleged medical reasons for abandoning the study, a lack of personal motivation may have contributed to our dropout rate at 3 months after the end of the intervention. Thus, the results must be interpreted with caution. A larger sample size and the introduction of motivational strategies may result in a greater degree of adherence to the intervention and may provide more conclusive results. Future studies in this field should specifically address and measure treatment adherence during the intervention period. In addition, it may be desirable to include a more comprehensive and extended follow-up period in future research in this field. In addition, we wish to draw attention to the fact that men were excluded from this RCT and, thus, their response to the intervention may be different. Finally, data collection took place in 2013–2014. Although it is unlikely that health promotion interventions in primary care in Spain have changed significantly since then, the data are not recent.

## 5. Conclusions

The impact of a health promotion intervention to decrease ST in the population of women with FM and moderate obesity was very limited. The results show a need for more experimental studies aimed at reducing ST in the population of patients with FM. However, we recommend that future interventions are tailored to the patients’ needs and characteristics, are supervised regularly by a healthcare professional, and integrate group activities with the aim of increasing motivation, and professional and social support.

## Figures and Tables

**Table 1:** Sociodemographic characteristics between the groups at baseline.

KERRYPNX	Control Group (*n* = 38)	Intervention Group (*n* = 46)	Total (*n* = 84)	*p* Value
Age (mean (SD))	57.42 (5.80)	54.21 (8.44)	55.48 (7.6)	0.472 ^a^
Employment status	Freq (%)	Freq (%)	Freq (%)	0.501 ^b^
Housewife	3 (7.9%)	8 (17.4%)	11 (12.5%)	
Unemployed	7 (18.4%)	8 (17.4%)	15 (17%)	
Unemployed due to disability	6 (15.8%)	8 (17.4%)	14 (15.9%)	
Employed	15 (39.5%)	16 (34.8%)	31 (35.2)	
Retired	6 (15.8%)	4 (8.7%)	10 (11.4%)	
No answer	1 (2.6%)	2 (4.3%)	1 (1.1%)	
Marital status	Freq (%)	Freq (%)	Freq (%)	0.206 ^b^
Single	2 (5.3%)	5 (10.9%)	7 (8%)	
Married	29 (76.3%)	29 (63%)	58 (65.9%)	
Widowed	0	3 (6.5)	3 (3.4%)	
Divorced	4 (10.5%)	8 (17.4)	12 (13.6%)	
No answer	3 (7.9%)	1 (2.2%)	4 (4.5%)	
Level of education	Freq (%)	Freq (%)	Freq (%)	<0.001 ^b^
University	1 (2.6%)	4 (8.7%)	5 (6%)	
Low/medium studies	33 (86.8%)	39 (84.7%)	72 (85.8%)	
No answer	4 (10.5%)	3 (6.5%)	7 (8.3%)	
Marshall Questionnaire	6.15 (3.46)	6.3 (4.3)	6.15 (3.46)	0.169 ^a^

^a^ ANOVA; ^b^ Chi-square test.

**Table 2:** Intergroup differences in anthropometric, clinical, and biochemical variables and activPAL™ values before and after the intervention and at 3-month follow-up.

	Pretest			Posttest			3-Month Follow Up			
	CG (*n* = 38)	IG (*n* = 46)	*p* ^a^	CG (*n* = 21)	IG (*n* = 31)	*p* ^a^	CG (*n* = 15)	IG (*n* = 19)	*p* ^a^	*p* ^b^
	Mean (SD)	Mean (SD)		Mean (SD)	Mean (SD)		Mean (SD)	Mean (SD)		
BMI (kg/m^2^)	28.67 (2.67)	30.13 (2.8)	0.017	29.04 (3.31)	30.0 (3.31)	0.138	29.67 (2.93)	29.21 (3.55)	0.108	0.000
Abdominal perimeter (cm)	102.75 (9.27)	103.03 (8.87)	0.887	100.4 (7.2)	104.6 (9.2)	0.151	96.5 (20.1)	102.4 (11)	0.493	0.687
Tricipital fold (cm)	33.95 (5.20)	28.51 (4.71)	<0.001	32.9 (5.9)	30.5 (5.08)	0.099	39.13 (17.7)	29.5 (5.3)	0.207	0.688
Systolic blood pressure (mmHg)	126.95 (16.11)	124.09 (14.56)	0.396	127.26 (13.8)	120.5 (10.5)	0.429	115.8 (16)	119 (14.9)	0.300	0.890
Diastolic blood pressure (mmHg)	78.82 (11.23)	79.50 (9.72)	0.766	77.94 (10.2)	72.6 (8.7)	0.073	80.8 (12.6)	72.2 (10.1)	0.058	0.417
Cholesterol (mg/dL)	215.58 (35.25)	219.67 (35.98)	0.602	230 (44.4)	233 (35.3)	0.877	209.7 (50.7)	208.5 (56.8)	0.816	0.444
HDL (mg/dL)	59.76 (13.38)	61.50 (11.89)	0.532	66 (22.9)	56.5 (6.3)	0.295	73.7 (29.3)	208.5 (56.8)	0.245	0.646
LDL (mg/dL)	128.81 (32.69)	137.07 (34.41)	0.270	133 (32.3)	153.1 (34.8)	0.429	111.6 (53.3)	116 (73.7)	0.611	0.761
Triglycerides (mg/dL)	142.32 (59.39)	119.72 (46.22)	0.053	160.5 (65.4)	142.1 (73.8)	0.433	124.7 (35.4)	125.75 (24.67)	0.054	0.161
Glycemia (mg/dL)	96.18 (15.66)	88.2 (10.04)	0.006	97.1 (18.7)	92.3 (9.3)	0.162	96.5 (23.9)	93.5 (12.9)	0.531	0.584
METs	37.5 (7.6)	36.1 (3.3)	0.522	35.31 (2.27)	35.88 (3.49)	0.400	35.50 (2.53)	36.05 (3.12)	0.062	0.483
Steps	8373.1 (2820.5)	9492.16 (5857.2)	0.309	9059 (3731.6)	8053.4 (3428.5)	0.187	8161.35 (3155.74)	9539.46 (5822.31)	0.675	0.000
Sitting time (hours)	10.08 (2.7)	10.9 (2.4)	0.157	10.9 (2.5)	10.7 (2.8)	0.243	10.50 (2.84)	10.31 (2.42)	0.048	0.413
Change position	46.49 (13.88)	49.14 (14.77)	0.424	46.19 (9.80)	42.67 (12.86)	0.029	47.25 (14.25)	48.56 (15.24)	0.587	0.891

^a^ Kruskal–Wallis test; ^b^ Friedman test: interaction *p*-value (group x time); CG: Control group; IG: Intervention group.

**Table 3:** Intragroup differences in the intervention and the control group at baseline and 3-month follow-up.

	Baseline CG	3-Month Follow-Up CG	*p* ^a^	Baseline IG	3-Month Follow-Up IG	*p* ^a^
	Mean (SD)	Mean (SD)		Mean (SD)	Mean (SD)	
BMI (kg/m^2^)	28.67 (2.67)	29.67 (2.93)	0.001	30.13 (2.8)	29.21 (3.55)	<0.001
Abdominal Perimeter (cm)	102.75 (9.27)	96.5 (20.1)	0.865	103.03 (8.87)	102.4 (11)	0.618
Tricipital Fold (cm)	33.95 (5.20)	39.13 (17.7)	0.124	28.51 (4.71)	29.5 (5.3)	0.913
Systolic Blood Pressure (mmHg)	126.95 (16.11)	115.8 (16)	0.184	124.09 (14.56)	119 (14.9)	0.968
Diastolic Blood Pressure (mmHg)	78.82 (11.23)	80.8 (12.6)	0.421	79.50 (9.72)	72.2 (10.1)	0.018
Cholesterol (mg/dL)	215.58 (35.25)	209.7 (50.7)	0.672	219.67 (35.98)	208.5 (56.8)	1.000
HDL (mg/dL)	59.76 (13.38)	73.7 (29.3)	0.248	61.50 (11.89)	208.5 (56.8)	0.197
LDL (mg/dL)	128.81 (32.69)	111.6 (53.3)	0.398	137.07 (34.41)	116 (73.7)	0.655
Triglycerides (mg/dL)	142.32 (59.39)	124.7 (35.4)	0.063	119.72 (46.22)	125.75 (24.67)	0.109
Glycemia (mg/dL)	96.18 (15.66)	96.5 (23.9)	0.735	88.2 (10.04)	93.5 (12.9)	0.713
METs	37.5 (7.6)	35.50 (2.53)	0.021	36.1 (3.3)	36.05 (3.12)	0.158
Steps	8373.1 (2820.5)	8161.35 (3155.74)	0.903	9492.16 (5857.2)	9539.46 (5822.31)	0.969
Sitting Time (hours)	10.08 (2.7)	10.50 (2.84)	0.382	10.9 (2.4)	10.31 (2.42)	0.084
Change Position	46.49 (13.88)	47.25 (14.25)	0.230	49.14 (14.77)	48.56 (15.24)	0.887

^a^ Wilcoxon test; CG: Control group, IG: Intervention group.

## Data Availability

The data presented in this study are available on request from the corresponding author. The data are not publicly available due to specific requirements from the clinical research ethics committee that reviewed and approved this investigation.
